# A Simple Eco-Friendly HPLC-PDA Method for the Simultaneous Determination of Paclitaxel and Seliciclib in Plasma Samples for Assessing Their Pharmacodynamics and Pharmacokinetics in Combination Therapy for Uterine Sarcoma

**DOI:** 10.3390/medicina60101601

**Published:** 2024-09-29

**Authors:** Amsha S. Alsegiani, Sarah Alrubia, Ibrahim A. Darwish

**Affiliations:** Department of Pharmaceutical Chemistry, College of Pharmacy, King Saud University, P.O. Box 2457, Riyadh 11451, Saudi Arabia; aalsegiani@ksu.edu.sa (A.S.A.);

**Keywords:** uterine sarcoma, paclitaxel, seliciclib, combination therapy, HPLC-PDA, green analytical chemistry, rapid and efficient analysis

## Abstract

*Background/Objectives*: Uterine sarcoma, a rare cancer originating in the smooth muscle of the uterus, exhibits high rates of recurrence and metastasis. It represents one of the most challenging types of cancer due to its chemorefractory nature, showing little response to conventional chemotherapy methods and displaying a relative survival rate of 30–40%. A potentially promising approach for treating uterine sarcoma involves combination therapy with paclitaxel (PAC), a microtubule-targeting agent, and seliciclib (SEL), a cyclin-dependent kinase inhibitor. SEL has been identified as a drug that can enhance the effectiveness of PAC through synergistic effects. To further refine this treatment strategy, an efficient analytical tool capable of simultaneously measuring the concentrations of PAC and SEL in blood plasma is needed. This tool would make it easier to study the pharmacokinetic interactions of potential drugs and assist in monitoring therapy when administering this combination treatment. Regrettably, a method meeting these specific requirements has not been documented in the existing literature. *Methods*: This article introduces the first HPLC technique employing a PDA detector to concurrently measure PAC and SEL levels in plasma. The methodology underwent validation in accordance with the ICH standards for validating bioanalytical methods. *Results*: The method exhibited linearity in the concentrations ranging from 0.8 to 100 µg mL^−1^ for both PAC and SEL. The limits of quantification were determined and found to be 1.34 and 1.25 µg mL^−1^ for PAC and SEL, respectively. All the other validation criteria conformed to the ICH validation standards. The HPLC-PDA method was successfully employed to quantify both PAC and SEL in plasma samples with a high level of reliability (in terms of accuracy and precision). The eco-friendliness of the approach was verified using three thorough assessments. This technique serves as a valuable asset in establishing the correct dosage and administration schedule for the combined treatment involving PAC and SEL, ensuring the desired therapeutic effects and safety in managing uterine sarcoma. *Conclusions*: The proposed HPLC-PDA method is the first reliable and eco-friendly method developed to simultaneously determine PAC and SEL in high-throughput plasma samples in clinical laboratories.

## 1. Introduction

Uterine sarcoma is a rare cancer that develops in the smooth muscle of the uterus with high rates of recurrence and metastasis [1]. It has two main histological malignancy types: uterus leiomyosarcoma (LMS) and mixed mesodermal tumors (MMTs). Many studies have reported that the 5-year relative survival rate for patients with uterine sarcomas is 30–40% [2,3]. Accordingly, uterine sarcomas are considered the most chemorefractory type of cancer, which means they do not respond to chemotherapy [4,5]. There are no recognized standard treatments for uterine sarcomas currently. Treating uterine sarcoma with a single chemotherapeutic agent has shown a lower efficacy than is desirable [6]. Paclitaxel (PAC), the most common microtubule-targeting agent (MTA), is one of the mono-chemotherapies that has been used; however, its response is unexpected, and using it alone is not recommended [7]. However, when used in combination, the results have been promising, and several retrospective studies have reported a potential benefit of combination chemotherapy in uterine sarcoma treatment [1,8,9].

A combination of the chemotherapies used in uterine sarcoma treatment has been recently extensively studied, and the results have demonstrated their combined potential to enhance the clinical effectiveness of the treatment [6]. A variety of medications with distinct molecular actions have been merged to enhance synergy, decrease therapeutic dosages, mitigate the development of multidrug resistance, and diminish the potential for overlapping toxicities [10,11]. Many preclinical studies have reported that some cyclin-dependent kinase (CDK) inhibitors form the most successful combinations with MTAs [5,12]. A combination of PAC and seliciclib (SEL), a promising CDK drug, has been reported in one preclinical study to have potential synergistic effects in treating uterine sarcoma [5]. It is suggested that SEL can enhance the efficacy of PAC by further disrupting the cell cycle, improving apoptotic effects, and promoting cancer cell death [5]. 

PAC has been approved as a first-line treatment for numerous types of cancer, such as breast, ovarian, gastric, and non-small cell lung cancer, as well as Kaposi sarcoma [13]. It inhibits cell replication during the mitotic phase of cell division by binding to tubulin, promoting the polymerization of the tubulin dimers, and subsequently stabilizing microtubules. Furthermore, PAC induces apoptosis in cancer cells by binding to the Bcl-2 protein, distorting mitotic spindles and leading to chromosome fragmentation [14,15]. On the other hand, the SEL drug displays a high efficiency and selectively inhibits CDK 2, 7, and 9 at their ATP binding sites [16,17]. SEL’s antiproliferative and apoptotic effects are controlled by several molecular mechanisms, including the inhibition of RNA polymerase II-dependent transcription, the downregulation of proliferative and survival proteins, the upregulation of p53, the decrease in Rb phosphorylation, and the modulation of E2F transcriptional activity [5,18,19]. 

Administering a combination of anticancer drugs with different pharmacokinetics, like PAC and SEL, is still a big challenge as it needs a convenient analytical method that is able to accurately and simultaneously quantify their concentrations in plasma samples [10]. The availability of such a method would be very important in refining the findings regarding the synergistic chemotropic effects of the PAC/SEL combination and supporting their pharmacokinetics and therapeutic drug monitoring. As evident from an extensive literature review, there are analytical methods that are available for the individual quantification of PAC [20] or SEL in plasma samples [21]; however, no method currently exists for simultaneously quantifying both PAC and SEL in plasma. Therefore, this study aims to pioneer a new approach for concurrently assessing PAC and SEL levels in plasma samples. The development of such a method would offer valuable insights into refining the synergistic chemotherapeutic effects of PAC and SEL in combination for treating uterine sarcoma, supporting their pharmacokinetics, possible drug–drug interactions, and therapeutic drug monitoring. Ultimately, this technique could deepen our understanding of the therapeutic benefits of combining PAC and SEL to enhance the treatment of uterine sarcoma patients. 

This work introduces a straightforward and environmentally conscious HPLC-PDA technique that allows for the concurrent quantification of PAC and SEL in plasma samples, marking an initial advancement in this area. This technique enables the measurement of both PAC and SEL at concentrations as low as 1.34 and 1.25 µg mL^−1^ for PAC and SEL, respectively. This level of sensitivity enables the quantification of both PAC and SEL in plasma samples after the administration of their recommended therapeutic doses. Following the International Council for Harmonization (ICH) guidelines for bioanalytical method validation and study sample analysis [22], the method underwent validation. This process verified the method’s suitability for its designated use. Furthermore, the eco-friendliness/greenness of the method was confirmed by three different metric tools. 

## 2. Experimental Procedure

### 2.1. Apparatus and Materials

HPLC system manufactured by Shimadzu Corporation in Kyoto, Japan was outfitted with an auto-sampler (SIL-30AC) and photodiode array (PDA) detector (LC-20AD) set at 230 nm. The instrument was controlled and data were acquired using LCsolution software (version 1.25), provided with the HPLC system. A pH meter (pH 211: Hanna, Nusfalau, Romania) was used to adjust the pH of buffer solutions. A Heidolph vortex mixer (D-91126; Schwabach, Germany) was used to mix standards with plasma samples. A Sigma centrifuge (1-15PK: Sigma, Osterode am Harz, Germany) was used for the precipitation of plasma proteins from plasma samples. Standard materials of SEL, PAC, and linifanib (LIN) were acquired from LC Laboratories in Woburn, MA, USA, with purities exceeding 99%. Zorbax Eclipse Plus C18 HPLC column (150 mm length × 4.6 mm internal diameter, 5 µm particle size) was a product of Agilent (Santa Clara, CA, USA). A guard column manufactured by Macherey-Nagel GmbH & Co. (Düren, Germany) was connected prior to the analytical column being used. HPLC-grade solvents were purchased from Merck (Darmstadt, Germany). All other materials were of analytical grade. Human plasma sourced from the Blood Bank of the King Khaled University Hospital in Riyadh, Saudi Arabia was preserved in a freezer at −20 °C until the analysis was conducted.

### 2.2. Preparation of Standard Solutions and Quality Control Samples

Accurate amounts (10 mg) of each PAC, SEL, and LIN (internal standard: IS) were separately transferred to 10 mL calibrated flasks. The materials were then dissolved in ~8 mL of acetonitrile, and the volumes were topped off to reach 10 mL with the same solvent. The concentration of these stock solutions was 1.0 mg mL^−1^. Then, these solutions were diluted with acetonitrile to prepare working standard solutions with concentrations of 50 µg mL^−1^ for PAC and SEL and 10 µg mL^−1^ for LIN. The calibration samples were prepared by spiking PAC, SEL, and LIN (IS) to blank human plasma, resulting in final concentrations of PAC and SEL ranging from 1.6 to 200 µg mL^−1^ and a constant concentration of IS at 10 µg mL^−1^ in all solutions. The samples were combined with an equivalent amount of methanol, vortexed for 30 s, and subsequently centrifuged at 13,000 rpm for 10 min utilizing the Eppendorf Himac Centrifuge from Hamburg, Germany. The resultant supernatants were filtered by aspiration through syringes fitted with 0.2 µm Millipore filters. For analysis, each sample (10 µL) was introduced into the HPLC system. Quality control (QC) samples were likewise formulated at three distinct concentration tiers: low, medium, and high. The concentrations of PAC and SEL were set at 5, 50, and 80 µg mL^−1^. These QC samples were prepared following the procedures used to prepare the calibration standards. The samples were kept in the refrigerator until they were required for analysis. Analysis of the samples took place on various days, with daily evaluations of the system suitability parameters.

### 2.3. HPLC Conditions and Statistical Analysis

The chromatographic resolutions were performed using a mobile phase composed of acetonitrile and an acetate buffer solution (pH 5) at a ratio of 50:50 (*v*/*v*). The mobile phase was delivered in isocratic mode at a flow rate of 1.0 mL min^−1^. Aliquots (10 µL) of the sample solutions (PAC and SEL standards, blank plasma, blank plasma spiked with LIN (IS), or blank plasma spiked with both PAC and SEL) were injected into the HPLC system by the autosampler. The relation between the peak area ratios (peak area of PAC and SEL to that of LIN) was plotted as a function of PAC and SEL. Calibration curves were generated, which showed the relationship of the peak area ratio (on the *Y*-axis) and the concentrations (on the *X*-axis). The regression equations were derived and used to determine both PAC and SEL in the samples.

Statistical analysis was conducted using Microsoft Excel software, specifically Microsoft Office 365 version 2021 from Microsoft Corporation in Redmond, WA, USA. Data were expressed as mean ± standard deviation (SD) or relative standard deviation (RSD). Regression analysis of the calibration curves for the HPLC-PDA method was performed utilizing the integrated Data Analysis Package in Excel software, with a significance level set at *p* < 0.05. This analysis encompassed the computation of various parameters, including the intercept, slope, correlation coefficient, and variance.

### 2.4. Validation of HPLC-PDA Method

The HPLC-PDA method proposed in this study underwent a thorough validation procedure in accordance with the International Council on Harmonisation (ICH) guidelines for bioanalytical method validation and study sample analysis, M10 [22]. This validation process was designed to confirm the method’s appropriateness in terms of linearity, sensitivity, precision, accuracy, selectivity, and ruggedness for quantifying both PAC and SEL in plasma samples.

#### 2.4.1. Assessment of Linearity and Sensitivity

To evaluate linearity, three distinct calibration curves were created, from which regression equations and their determination coefficients were determined. The values of these coefficients were used as measures to assess the degree of the method’s linearity. The method’s sensitivity was assessed through the determination of the limit of detection (LOD) and limit of quantification (LOQ), calculated using the formula: LOD or LOQ = × SDa/b, with × being 3.3 for LOD and 10 for LOQ. Here, SDa represents the standard deviation of the calibration line intercept, while b denotes its slope.

#### 2.4.2. Determination of Precision and Accuracy

Intra-assay precision and accuracy were assessed by testing six replicate samples of each QC sample at three distinct levels (low, medium, and high) in a single batch. Similarly, inter-assay precision and accuracy were determined by analyzing three replicate samples of each QC sample at every level over three consecutive days. Precision was evaluated based on the relative standard deviation percentage (RSD, %) calculated from the repeated measurements. Accuracy was expressed as the recovery percentage (recovery, %), determined by comparing the measured concentrations with the designated concentrations.

#### 2.4.3. Assessment of Method Ruggedness

Evaluating the robustness of the method involved utilizing the same operating conditions for analyzing both PAC and SEL over three consecutive days by two different analysts. The results were presented as the relative standard deviation (RSD, %), offering insight into the variability within the data.

### 2.5. Assessment of Eco-Friendliness/Greenness of HPLC-PDA Procedures

The eco-friendliness/greenness of HPLC-PDA procedures was assessed by 3 different tools. These tools were the analytical eco-scale (AES) [23], green analytical procedure index (GAPI) [24], and analytical greenness (AGREE) [25].

AES tool evaluates the environmental effect of four parameters intrinsic to the analytical processes. Each parameter incurs penalty points (PPs) based on its adverse effects on the environment. The tool’s instructions and guidelines provide specific information on the calculation of these PPs. The total PPs accrued are then deducted from 100, representing the highest eco-friendliness score for the process. Based on the resulting variance, the AES tool classifies procedures based on their eco-friendliness into three tiers: green (total score > 75 points), moderately green (total score of 50–75 points), and inadequately green (total score < 50 points).

The GAPI tool estimates the overall analytical process through 15 parameters (1–15), covering activities from sample collection to waste generation/treatment post-analysis. These parameters are visually represented in a pictogram comprising 15 segments, with each segment symbolizing an assessment parameter designated a specific color (green, yellow, or red). Green signifies an environmentally sustainable process, while red signifies a procedure lacking environmental friendliness. Specific instructions on assigning colors to respective parameters are elucidated in the tool’s guidelines.

The AGREE tool is user-friendly and thorough software designed for assessing the environmental sustainability of analytical methods based on the 12 principles of green analytical chemistry (GAC) specified in the software guidelines. This tool automatically produces results presented in a circular pictogram format. Each segment of the pictogram is allocated a particular color, varying from intense green (=1) to deep red (=0), reflecting its influence on assay sustainability. The total score exhibited at the middle of the pictogram is computed as a fraction of unity.

## 3. Results and Discussion 

### 3.1. Strategy of Method Development 

Due to the promising and noteworthy clinical therapeutic advantages demonstrated by the combination of PAC and SEL, there is an immediate and pressing need to establish a convenient analytical technique for their simultaneous quantification in plasma. This methodology is necessary to refine clinical findings and empower researchers to delve into the pharmacodynamics and pharmacokinetics of PAC and SEL when administered concurrently. This becomes especially crucial given their possible synergies in treating uterine sarcomas. The results gleaned from these studies could ultimately support the enhancement and customization of PAC-SEL combination therapy, paving the way for personalized treatment strategies.

**Figure 1:**
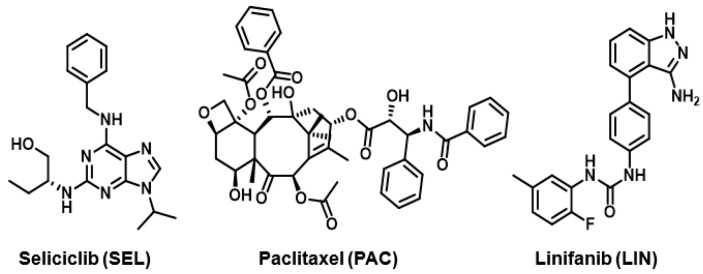
The chemical structures and abbreviations of seliciclib, paclitaxel, and linifanib.

**Figure 2:**
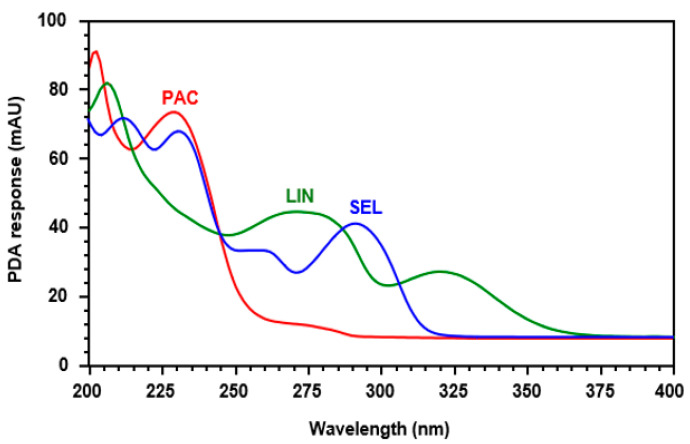
The UV absorption spectra of SEL, PAC, and LIN. These spectra were generated by the PDA detector of the HPLC system. The concentrations of SEL, PAC, and LIN solutions were 50, 10, and 100 µg mL^−1^, respectively.

Liquid chromatography coupled with tandem mass spectrometry (LC-MS/MS) is a highly effective analytical method extensively utilized in pharmaceutical and biomedical analyses [26]. However, it comes with significant drawbacks, including high acquisition and operational expenses, a need for advanced technical proficiency for efficient operation, time-consuming and labor-intensive optimization processes, and challenges in standardization. A viable alternative to LC-MS/MS is HPLC with PDA detection, which presents its own array of benefits such as its cost-effectiveness, simplicity, accessibility, robustness, stability, regulatory adherence, and compatibility with diverse sample matrices [27]. Given the chromophoric chemical structures of PAC and SEL (depicted in Figure 1), it is anticipated that they possess notable UV light absorption capabilities with unique absorption bands but differing maximum absorption wavelengths. This expectation was confirmed by the UV absorption spectra of both compounds (Figure 2). Hence, the objective of this research was to utilize the UV absorption properties of these compounds in establishing an HPLC-PDA technique for quantifying them in plasma samples. Expanding on the achievements of prior research endeavors undertaken by our team, which concentrated on devising simple non-extractive methods for preparing plasma samples before HPLC analyses of specific pharmaceuticals [28,29], a comparable non-extractive approach was applied in crafting the HPLC-PDA methodology outlined in this investigation.

### 3.2. Optimization of the Chromatographic Conditions

#### 3.2.1. Mobile-Phase Composition

Chromatographic conditions were optimized to achieve the best resolution of PAC, SEL, and LIN (IS). In the beginning, isocratic elution was investigated by utilizing a C18 column (250 mm length × 3.9 mm i.d., 5 μm particle diameter) linked to a guard column, maintaining a consistent column temperature of 25 ± 2 °C. Previous studies involving HPLC analyses of PAC have demonstrated that the mobile phase should contain ≥50% organic solvents to obtain a shorter retention time [30,31]. Additionally, buffer solutions with a pH around five are commonly used in the mobile phase to improve the selectivity, separation, peak shape, and signal response [32]. To the best of our knowledge, no HPLC method has been reported for SEL; this is the first study that aims to develop an HPLC method for SEL analysis. Based on these facts, initial separation trials were performed employing a mobile phase consisting of a methanol:acetate buffer at a pH of five (80:20, *v*/*v*) and a flow rate of 1 mL min^−1^. Employing these conditions, PAC was eluted at a relatively short time (4.22 min); however, its peak overlapped with that of SEL, which eluted at 4.31 min. Afterward, the methanol content of the mobile phase was changed to 70% to improve the resolution of PAC and SEL within an appropriately short run time and reduce the overlap of peaks between PAC and SEL. This alteration resulted in the conversion of the sequence of elution with a better, but not satisfactory, resolution; however, the run time was required. Subsequently, the acetate buffer was replaced with a formate buffer at a pH of five while keeping the methanol content at the same value (70%) to compare these new conditions with the previous conditions containing the acetate buffer. Under these conditions, a distinct separation between PAC and SEL was achieved within 10 min, with SEL being the first to emerge; the retention times of SEL and PAC were 4.49 and 8.9 min, respectively. Since the targeted HPLC-PDA method was being developed to simultaneously analyze SEL and PAC in plasma samples, it was necessary to relatively elongate the retention time of SEL to avoid any potential development with the early eluted plasma components. To achieve this goal, the methanol content was decreased to 65% instead of 70%. This modification resulted in a convenient retention time for SEL (5.7 min); however, the retention time of PAC was excessively undesirable and extended to 16.21 min. To address this issue, methanol was replaced with acetonitrile while keeping the formate buffer composition of the mobile phase. The initial content of acetonitrile was 40%, which was lower than that of methanol (65%) because acetonitrile has a higher elution power than methanol [33]. This alteration led to a more conveniently rapid elution of both SEL and PAC (at 5.01 and 7.42 min, respectively); regrettably, it caused a decline in the peak intensity for both compounds. Accordingly, the acetate buffer system was used instead of the formate and tested with different acetonitrile:acetate buffer ratios (70:30, 60:40, and 50:50, *v*/*v*). The best separation was achieved at a ratio of 50:50 (*v*/*v*) at which the retention times of SEL and PAC were 5.96 and 11.78 min, respectively (Table 1). 

#### 3.2.2. Column Packing and Dimensions 

To select the most suitable column, various additional reversed-phase columns with distinct dimensions and particle sizes were assessed. The columns that underwent assessments included the Shim-pack VP-ODS C18 (150 × 4.6 mm, 5 µm), Waters Spherisorb S5 C8 (150 × 4.6 mm, 4 µm), µBondapak C18 (300 × 3.9 mm, 5 µm), and BDS Hypersil C18 (250 mm × 4.6 mm, 5 µm). None of these columns yielded significant enhancements in the resolution of PAC and SEL within the specified chromatographic conditions. 

#### 3.2.3. Internal Standard and Detection Wavelength 

The UV spectra of both PAC and SEL (Figure 2) demonstrate their high absorption intensity in the ranges of 200–255 and 200–315 nm for PAC and SEL, respectively. Considering the demand for a sensitive HPLC method to measure both PAC and SEL, the PDA detector was set at 230 nm, at which point both PAC and SEL showed significantly high light absorption levels. A previous study conducted in our laboratory, involving the development of analytical methods for different chemotherapeutic drugs, demonstrated that linifanib (LIN) and other compounds have UV absorption capabilities at 230 nm [34], enabling their detection at the same detection wavelength of both PAC and SEL (Figure 2). These compounds were tested for their potential use as an internal standard for the development of the HPLC-PDA method for PAC and SEL. Among these tested compounds, LIN was the most appropriate as it was clearly separated under the same established chromatographic conditions for PAC and SEL. As a result, LIN was chosen as the internal standard (IS) for the development of the HPLC-PDA method outlined in this research.

#### 3.2.4. Peak Purity and System Suitability 

**Figure 3:**
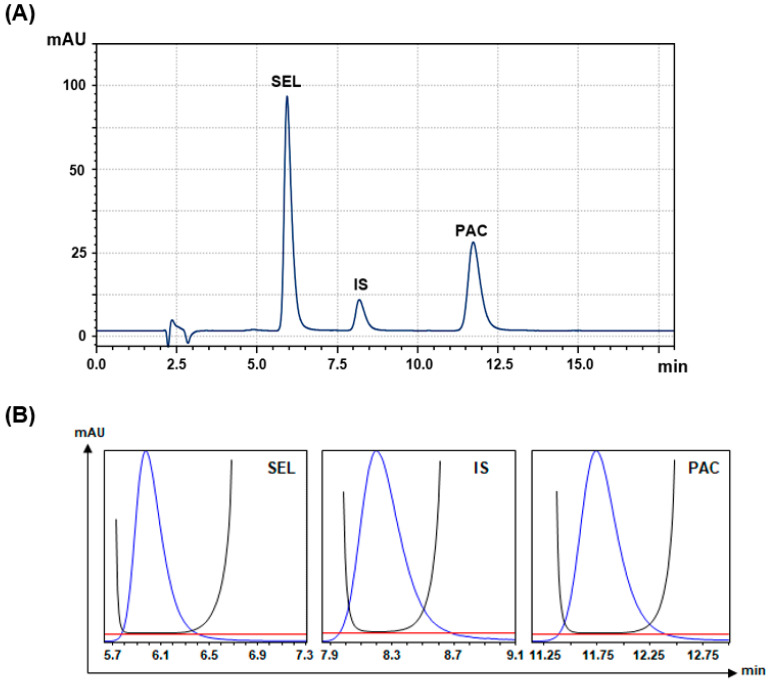
Panel (**A**): a representative chromatogram of standard solution containing SEL, PAC, and IS. The concentrations of SEL, PAC, and IS were 50, 50, and 10 µg mL^−1^, respectively. mAU is the detector response in millivolts as arbitrary units. Panel (**B**): the purity plots of the peaks in the chromatogram presented in Panel (**A**). The red, blue, and black curves are the threshold (zero) lines, peaks, and purity curves, respectively.

Under these refined chromatographic conditions, SEL, IS, and PAC had clearly separated, sharp, and symmetric peaks eluted at 5.79, 8.23, and 11.78 min, respectively (Figure 3A). The purity plots of the peaks were generated (Figure 3B), and the corresponding numerical values were computed and are presented in Table 2. In the peak purity plots (Figure 3B), the purity curves do not rise above the threshold lines. This indicates that there are no spectral differences beyond noise contributions and implies the presence of only one compound in the chromatographic peak. The peak purity indices and single point thresholds of all peaks were approximately one, confirming the purity of the peaks and the absence of any co-eluted compounds with the analytes of interest. 

The system suitability parameters were calculated and are presented in Table 2 to confirm the chromatographic system performance. All system suitability factors are within the acceptance requirements, suggesting the proposed method showed excellent levels of selectivity and baseline separation.

### 3.3. Results of Validation of HPLC-PDA Method

Method validation was conducted following ICH guidance for bioanalytical method validation for all parameters [22]. The validation parameters were evaluated on the drug reference material and plasma samples. Linearity, sensitivity (LOD and LOQ), accuracy, precision, stability, selectivity, carryover, and ruggedness were assessed.

#### 3.3.1. Linearity 

**Figure 4:**
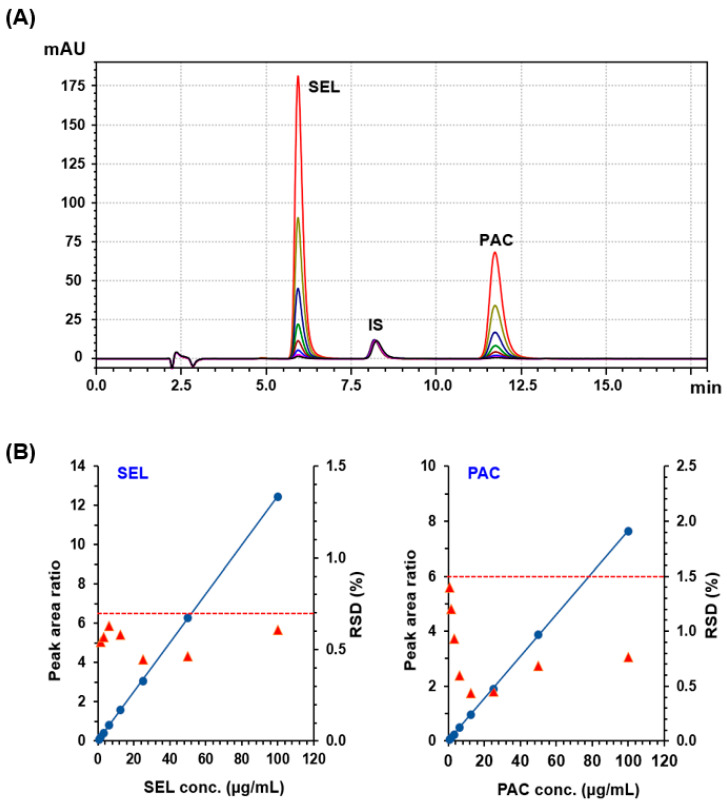
Panel (**A**): Overlaid chromatograms of standard solutions containing varying concentrations (0.78–100 µg mL^−1^) of SEL and PAC. All solutions contained a fixed concentration of IS (10 µg mL^−1^). Panel (**B**): the calibration curves (●, on the left axis) and precision profiles, expressed as RSD % (▲, on the right axis), of the HPLC-PDA method for the simultaneous determination of SEL and PAC.

A series of varying concentrations of PAC and SEL were performed under the specified conditions. The overlaid chromatograms of standard solutions containing varying concentrations are shown in Figure 4A. The ratios of peak areas of each analyte to the internal standard (IS) were graphed against the respective analyte concentrations to construct the calibration curves, depicted in Figure 4B. These data were employed in a linear regression analysis, revealing strong linear correlations with high correlation coefficient (r^2^) values for both analytes. Additionally, minimal intercept values indicated that the developed method exhibited significant levels of sensitivity and linearity. The linear fitting equations of both PAC and SEL are as follows:

For PAC: Y = 0.0018 + 0.0764X (r^2^ = 0.9999)

For SEL: Y = 0.0065 + 0.1245X (r^2^ = 0.9999)

where Y and X are the peak area ratio and analyte concentration, respectively. The linearity and statistical parameters of each analyte are summarized in Table 3. 

#### 3.3.2. The Limits of Detection Quantification 

According to the method requirements, the limits of detection (LOD) and quantification (LOQ) were determined for each analyte, as detailed in Table 3. The calculated LOD values were 0.44 and 0.41 µg mL^−1^ for PAC and SE, respectively, whereas the LOQ values were 1.34 and 1.25 µg mL^−1^ for PAC and SEL, respectively. These results demonstrated the adequate sensitivity of the proposed HPLC-PDA method for quantifying the therapeutic concentrations of both compounds and allowed for the extension of the technique for a fundamental analysis. This conclusion relied on the fact that the stated plasma concentrations of PAC ranged from 4.5 to 5.7 µM (3.84 to 4.87 µg mL^−1^) with a maximum plasma concentration (C_max_) of 5.1 µM (4.35 µg mL^−1^). These concentrations were reached after the administration of a dose of Cremophor-diluted PAC of 175 mg/m^2^ given as a 3 h infusion [13]. For SEL, the reported maximum plasma concentration of SEL was 1.56 µg mL^−1^ after the administration of a dose of 600 mg of SEL and 4.48 µg mL^−1^ after the administration of a dose-limiting toxicity [35].

#### 3.3.3. Accuracy and Precision

The accuracy (recovery %) and precision (RSD %) results for the three QC sample concentrations (5, 50, and 80 μg mL^−1^) are compiled in Table 4. The intra-day results demonstrated that the recovery values for PAC and SEL ranged from 97.9% to 100.4%, while the inter-day recovery values ranged from 98.6% to 102.2%. The RSD values for both analytes regarding their intra-day precision varied between 0.6% and 1.8%, while for their inter-day precision, the values ranged from 0.6% to 1.5%. These findings indicate that the HPLC-PDA method proposed reaches acceptable levels of accuracy and precision as per the guidelines set forth by ICH for bioanalytical method validation [22].

#### 3.3.4. Ruggedness

Ruggedness was evaluated concerning the variations between analysts and across different days. The ruggedness outcomes are presented in Table 5, with recovery values spanning from 98.2 to 101.5% and RSD values staying below 1.5%, affirming the robustness and consistency of the method under consideration.

#### 3.3.5. Selectivity

**Figure 5:**
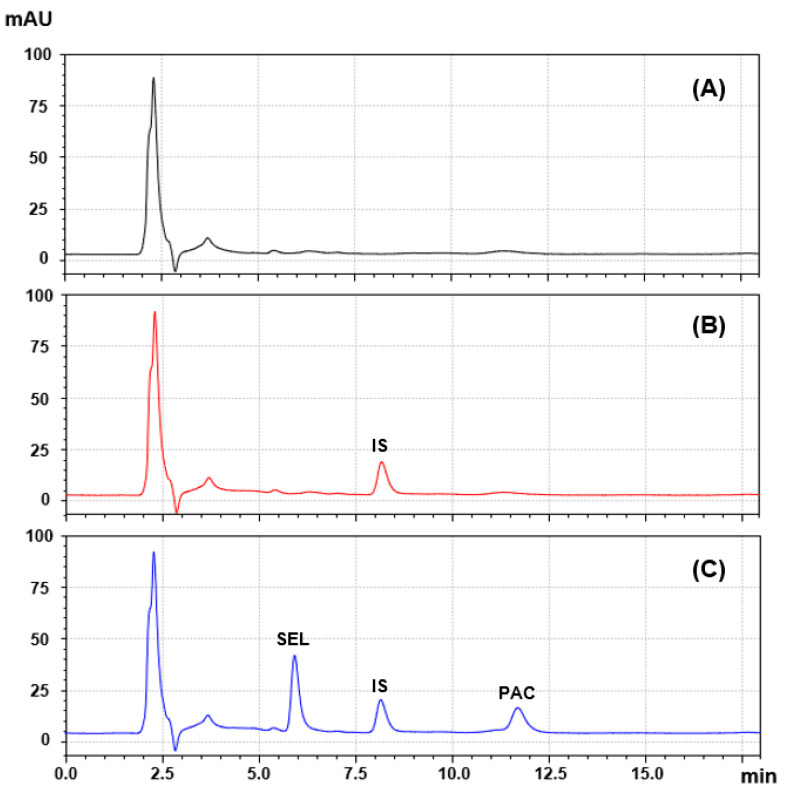
Representative chromatograms of blank (drug-free) human plasma (**A**), plasma spiked with IS at a concentration of 10 µg mL^−1^ (**B**), plasma spiked with IS, SEL, and PAC at concentrations of 10, 25, and 25 µg mL^−1^, respectively (**C**).

The specificity of the bioanalytical HPLC-PDA method under consideration was evaluated by analyzing blank human plasma (drug-free), plasma samples spiked with IS alone, and plasma samples spiked with PAC and SEL with IS. All obtained chromatograms are shown in Figure 5. No additional specific interference peaks were observed in blank human plasma for any of the analyte retention times, indicating an absence of interference. Furthermore, there were no instances of carryover effects detected for PAC, SEL, or the internal standard (IS) in the plasma samples. This was evidenced by the lack of peaks following the successive injections of the plasma samples containing the limit of quantification (LOQ) concentrations of both PAC and SEL. Therefore, this bioanalytical method offers the absence of interference and high levels of selectivity and sensitivity.

### 3.4. Analysis of PAC and SEL in Plasma Pamples

Four QC concentrations (10, 20, 40, and 80 µg mL^−1^) were spiked with drug-free plasma to assess the practical applicability of the proposed HPLC-PDA method for plasma analysis. The concentration and recovery of these samples in plasma are represented in Table 6. The mean recovery for PAC and SEL was 100.3 ± 1.7% and 99.7 ± 1.7%, respectively. The concentrations of both PAC and SEL spiked in plasma samples were plotted as a function of those measured by the proposed HPLC-PDA method (Figure S3). The linear regression analysis of the results demonstrated strong correlations between the measured and spiked concentrations, as revealed by the high values of correlation coefficients of the obtained linear fitting equations:

For PAC: Y = 0.0.2417 + 1.0091X (r = 0.9992)

For SEL: Y = 0.0.6878 + 0.9794X (r = 0.9995)

where Y and X are the concentrations spiked in plasma and those measured by the HPLC-PDA method, respectively. Additionally, the values of the slopes of the correlation lines (~1) further indicated the high accuracy of the proposed method for quantifying PAC and SEL in plasma samples. These results confirm the validity of the proposed HPLC-PDA method for quantifying both PAC and SEL in plasma after their combined administration, enabling the extension of the potential method to further pharmacodynamic and pharmacokinetic studies.

### 3.5. Eco-Friendliness/Greenness of HPLC-PDA Procedures

In green analytical chemistry (GAC), eco-friendly analytical techniques are strongly encouraged during the analytical steps, from sample preparation to final sample determination [36]. This involves decreasing the use of dangerous chemicals, reducing waste production, and preserving energy. In this study, the greenness sustainability of the proposed HPLC-PDA method was assessed to provide a precise and comprehensive evaluation of the greenness of analytical procedures. Three different metrics tools were used to assess the greenness of the proposed methods: the AES, GAPI, and AGREE. The results obtained from these tools are discussed in the following sections.

#### 3.5.1. Analytical Eco-Scale (AES)

The outcomes derived from the AES tool are detailed in Table 7. Penalty points (PPs) were allocated to the solvent (acetonitrile) and reagent (ammonium acetate), with two and four PPs for the amount of acetonitrile and ammonium acetate, respectively. In terms of the hazard impact, the assigned PPs were one and two PPs for acetonitrile and ammonium acetate, respectively. The parameters of energy consumption by the instrument and occupational hazards received one PP because these parameters adhered to the tool’s instructions. The cumulative penalty points (PPs) of waste generation and management were five and three PPs, respectively. These points were assigned due to the HPLC-PDA method generating 1–10 mL of waste per sample without subsequent treatment. As a result, the HPLC-PDA process accrued a total of 18 PPs, leading to an eco-scale rating of 82 (a result of 100 minus 13). This elevated score signifies the remarkable environmental sustainability of the HPLC-PDA technique, in accordance with the criteria set by the AES tool.

#### 3.5.2. Green Analytical Procedure Index (GAPI)

**Figure 6:**
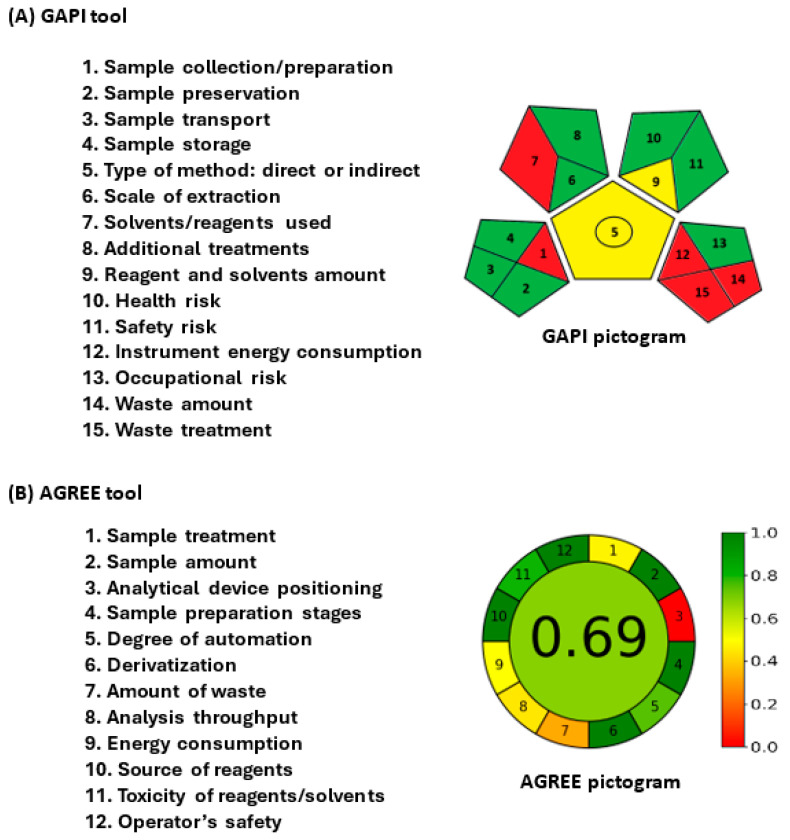
The evaluation of the greenness of the proposed HPLC-PDA method for the simultaneous determination of SEL and PAC by GAPI (**A**) and AGREE (**B**) tools. The evaluation parameters and pictograms are given on the left-hand and right-hand section of each panel.

The findings from the GAPI tool are showcased through a visual representation comprising 15 evaluation criteria (Figure 6A). Within these criteria, three (1, 7, 12, 14, and 15) are highlighted in red on the diagram, signaling their non-compliance with the GAC standards. Parameter 1 indicates that samples were gathered or prepared offline, while parameter 7 denotes the utilization of methanol for protein precipitation from plasma samples. While it is true that methanol can be toxic to human cells at elevated concentrations, its use in analytical procedures such as protein precipitation is commonly employed due to its effectiveness and efficiency. In the context of green chemistry, the key consideration is not only the potential toxicity of a solvent but also its overall environmental impact and sustainability. When used judiciously and in controlled amounts, methanol can still be considered a relatively safer option for protein precipitation compared to other solvents with higher toxicity profiles. Additionally, the green aspect of using methanol in this context may stem from factors such as its biodegradability, lower environmental persistence, and potential to be recycled. It is essential to emphasize that in green analytical chemistry, the goal is to minimize the overall environmental footprint of analytical procedures while ensuring the safety of both researchers and the environment. Through the proper handling, disposal, and consideration of the concentration used, methanol can be integrated into green analytical procedures responsibly. Parameter 15 highlights the lack of waste treatment for the assay. Parameter 5 is marked in yellow because the method is a direct approach used for quantitative purposes. Parameters 6 and 10 are also shown in yellow because the sample extraction was performed on a small scale, and the solvent poses a moderate health risk. The remaining parameters are displayed in green, indicating their full adherence to the eco-friendly procedure standards outlined in the GAPI tool guidelines. In summary, out of the fifteen parameters, eight (53.3%) are green, two (13.3%) are yellow, and five (33.3%) are red. These findings underscore the satisfactory environmental sustainability of the procedures employed in the proposed HPLC-PDA method.

#### 3.5.3. Analytical Greenness (AGREE)

The pictogram generated by the AGREE tool is displayed in Figure 6B. Parameter 1, related to sample handling, is marked in yellow due to the manual treatment of samples. Parameter 3, regarding device configuration (online or offline), is marked in red because the analysis on the HPLC system was conducted offline. Parameter 7 is indicated in a shade of reddish-yellow as the procedures generated more than 15 mL of waste in the mobile phase. Parameter 8 is represented in a darker shade of yellow due to the extended 15 min run time of the procedure. Parameter 9 is depicted in yellow based on the energy consumption of the HPLC system. The remaining parameters are depicted in green, indicating adherence to environmentally friendly practices. The overall score attained was 0.69 out of 1, signifying a high level of eco-friendliness for the proposed HPLC-PDA method.

## 4. Conclusions

This study introduces the first method for the simultaneous quantification of PAC and SEL in human plasma using the HPLC-PDA system. The major outcomes of the study can be summarized in the following key points. The method provides a valuable analytical tool with a high level of sensitivity to accurately quantify PAC and SEL in real plasma at concentrations as low as 1.34 and 1.25 µg mL^−1^, respectively. The sample pretreatment process does not involve laborious extraction procedures like time-consuming liquid–liquid or solid-phase extraction; it involves a very simple precipitation of plasma proteins using methanol. The extraction recovery ranged from 97.9% to 102.2% and from 98.6% to 100.4% for PAC and SEL, respectively. The complete chromatographic separation of PAC and SEL was achieved within a relatively short run time (~12 min), enabling the processing of many samples in a short time. The method demonstrated a high level of precision as the RSD values did not exceed 1.8%. The greenness assessment of the proposed HPLC-PDA method demonstrated that the method has minimal environmental impact while maintaining analytical performance parameters. The HPLC-PDA method can be utilized to refine the pharmacodynamic findings of combination therapy with PAC and SEL, support their pharmacokinetic profiling and therapeutic drug monitoring, and serve as a basis for future research investigating drug–drug interactions in humans.

## Figures and Tables

**Table 1:** A summary of the optimization of the mobile-phase composition for separation of SEL and PAC for the development of HPLC-PDA method for their simultaneous determination.

Organic Solvent/Buffer Solution	Organic Solvent: Buffer Ratio (%, *v*/*v*)	Retention Time (min)	
		SEL	PAC
Methanol			
Acetate buffer, pH 5	80:20	4.31	4.22
Acetate buffer, pH 5	70:30	8.05	9.10
Formate buffer, pH 5	70:30	4.49	8.90
Formate buffer, pH 5	65:35	5.70	16.21
Acetonitrile			
Formate buffer, pH 5	40:60	5.01	7.42
Acetate buffer, pH 5	70:30	3.52	4.01
Acetate buffer, pH 5	60:40	4.20	5.80
Acetate buffer, pH 5	50:50	5.96	11.78

**Table 2:** Peak purity profiling and chromatographic system suitability parameters of the HPLC-PDA method for the simultaneous determination of SEL and PAC.

Parameter	Value		
	SEL	PAC	IS
Peak purity			
Impurity detection	Not detected	Not detected	Not detected
Purity index	1.000000	1.000000	0.999997
Single point threshold	0.999887	0.998983	0.990865
Minimum purity index	113	1017	9132
System suitability			
Retention time of SEL (min)	5.97	11.78	8.23
Capacity factor (*K*′)	1.747	4.416	2.747
Separation factor (α)	1.710	1.586	1.593
Resolution factor (Rs)	5.080	5.600	4.579
Peak asymmetry factor	1.641	1.405	1.514
Number of theoretical plates/m	11,920	17,085	14,483

**Table 3:** Calibration parameters for the determination of PAC and SEL by the proposed HPLC-PDA method.

Parameter	Value	
	PAC	SEL
Linear range (µg mL^−1^)	0.8–100	0.8–100
Intercept (a)	0.0018	0.0065
Standard deviation of intercept (SD_a_)	0.01026	0.0155
Slope (b)	0.0764	0.1245
Standard deviation of slope (SD_b_)	0.0143	0.0014
Determination coefficient (r^2^)	0.9999	0.9999
Limit of detection (LOD, µg mL^−1^)	0.44	0.41
Limit of quantification (LOQ, µg mL^−1^)	1.34	1.25

**Table 4:** Accuracy and precision of the proposed HPLC-PDA method for the simultaneous determination of PAC and SEL at different concentration levels.

Drug/Concentration (μg mL^−1^)	Recovery (% ± RSD) ^a^	
	Intra-Assay, *n* = 3	Inter-Assay, *n* = 6
PAC		
5	100.2 ± 1.4	100.4 ± 1.2
50	97.9 ± 1.8	99.8 ± 0.6
80	99.4 ± 0.6	102.2 ± 1.5
SEL		
5	99.8 ± 1.4	98.6 ± 1.2
50	100.4 ± 1.2	100.4 ± 1.5
80	98.6 ± 1.4	100.2 ± 0.8

^a^ Values are mean of three determinations.

**Table 5:** Ruggedness of the proposed HPLC-PDA method for the simultaneous determination of PAC and SEL.

Parameters	Recovery (% ± RSD) ^*a*^	
	PAC	SEL
Analyst-to-analyst		
Analyst-1	98.8 ± 1.2	101.2 ± 1.4
Analyst-2	100.2 ± 0.6	99.4 ± 1.2
Day-to-day		
Day-1	101.5 ± 1.4	98.2 ± 1.5
Day-2	99.2 ± 0.8	101.1 ± 0.8
Day-3	100.2 ± 0.6	99.5 ± 1.2

*^a^* Values are the mean of three determinations.

**Table 6:** Determination of PAC and SEL in plasma samples by the proposed HPLC-PDA method.

Spiked Concentration (μg mL^−1^)	PAC		SEL	
	Found Concentration ^a^ (μg mL^−1^)	Recovery (%) ^a^	Found Concentration ^a^ (μg mL^−1^)	Recovery (%) ^a^
10	9.8	98.4	9.9	99.4
20	19.8	99.1	20.3	101.3
40	40.3	100.8	40.9	102.1
80	80.4	100.5	78.6	98.3
	Mean	99.7	Mean	100.3
	RSD	1.1	RSD	1.7

^a^ Values are mean of three determinations.

**Table 7:** Analytical eco-scale for assessing the greenness of the proposed HPLC-PDA method for the simultaneous determination of SEL and PAC.

Eco-Scale Score Parameters	Penalty Points (PPs)
Solvent/reagent	
Amount: acetonitrile (>10 mL per sample)	2
Ammonium acetate buffer (>10 mL per sample)	4
Hazards: acetonitrile	1
Ammonium acetate buffer	2
Instrument: Energy used (kWh per sample)	
HPLC-PDA	1
Occupational hazardous	
Analytical process hermetic	0
Emission of vapors and gases into the air	0
Waste	
Production (>10 mL per sample)	5
Treatment (No treatment involved)	3
Total PPs	18
Eco-Scale score	82

## Data Availability

All data are available from the corresponding author (idarwish@ksu.edu.sa).

## Supplementary Materials

The following supporting information can be downloaded at: https://www.mdpi.com/article/10.3390/medicina60101601/s1, Figure S1: Chromatograms obtained for separation of PAC (black) and SEL (pink) a mobile phase composed of methanol:buffer solution (pH 5) at different ratios. The injected concentrations of PAC and SEL were 10 and 50 µg mL^−1^, respectively; Figure S2: Chromatograms obtained for separation of PAC (black) and SEL (pink) a mobile phase composed of acetonitrile:buffer solution (pH 5) at different ratios. The injected concentrations of PAC and SEL were 10 and 50 µg mL^−1^, respectively; Figure S3: The correlations of concentrations of PAC and SEL spiked in plasma samples with those measured by the proposed HPLC-PDA method. The linear fitting equations and their correlation coefficients are given on the correlation lines.
