# Supplementary material for: A Simple Eco-Friendly HPLC-PDA Method for the Simultaneous Determination of Paclitaxel and Seliciclib in Plasma Samples for Assessing Their Pharmacodynamics and Pharmacokinetics in Combination Therapy for Uterine Sarcoma
Source: Medicina (Kaunas). 2024 Sep 29;60(10):1601. doi: 10.3390/medicina60101601 (PMC11509227; doi:10.3390/medicina60101601)
Supplement: Supplementary file 1 [file medicina-60-01601-s001.zip › medicina-3202650-supplementary.pdf]

Supplementary Materials

A Simple Eco-Friendly HPLC-PDA Method for the Simultaneous Determination of Paclitaxel and Seliciclib in Plasma Samples for Assessing Their Pharmacodynamics and Pharmacokinetics in Combination Therapy of Uterine Sarcoma

Amsha S. Alsegiani, Sarah Alrubia and Ibrahim A. Darwish *

Department of Pharmaceutical Chemistry, College of Pharmacy, King Saud University, PO Box 2457, Riyadh 11451, Saudi Arabia.

* Correspondence: Correspondence: Ibrahim A. Darwish, Prof.
Department of Pharmaceutical Chemistry, College of Pharmacy, King Saud University, PO Box 2457, Riyadh 11451, Saudi Arabia. Tel: +966114677348; Fax: +966114676220; E-mail: idarwish@ksu.edu.sa

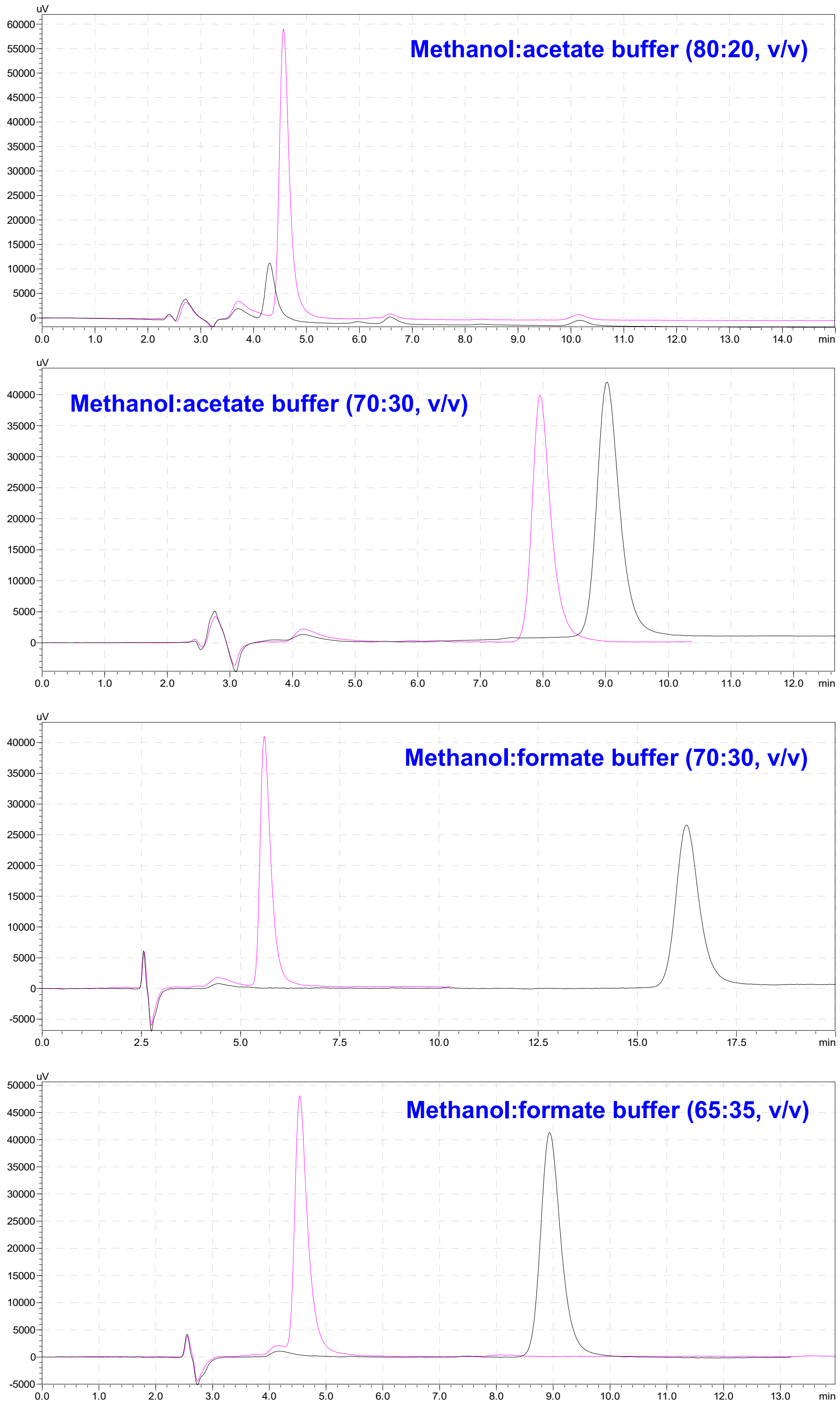

Figure S1: Chromatograms obtained for separation of PAC (black) and SEL (pink) a mobile phase composed of methanol:buffer solution (pH 5) at different ratios. The injected concentrations of PAC and SEL were 10 and 50 μg mL^{-1}, respectively.

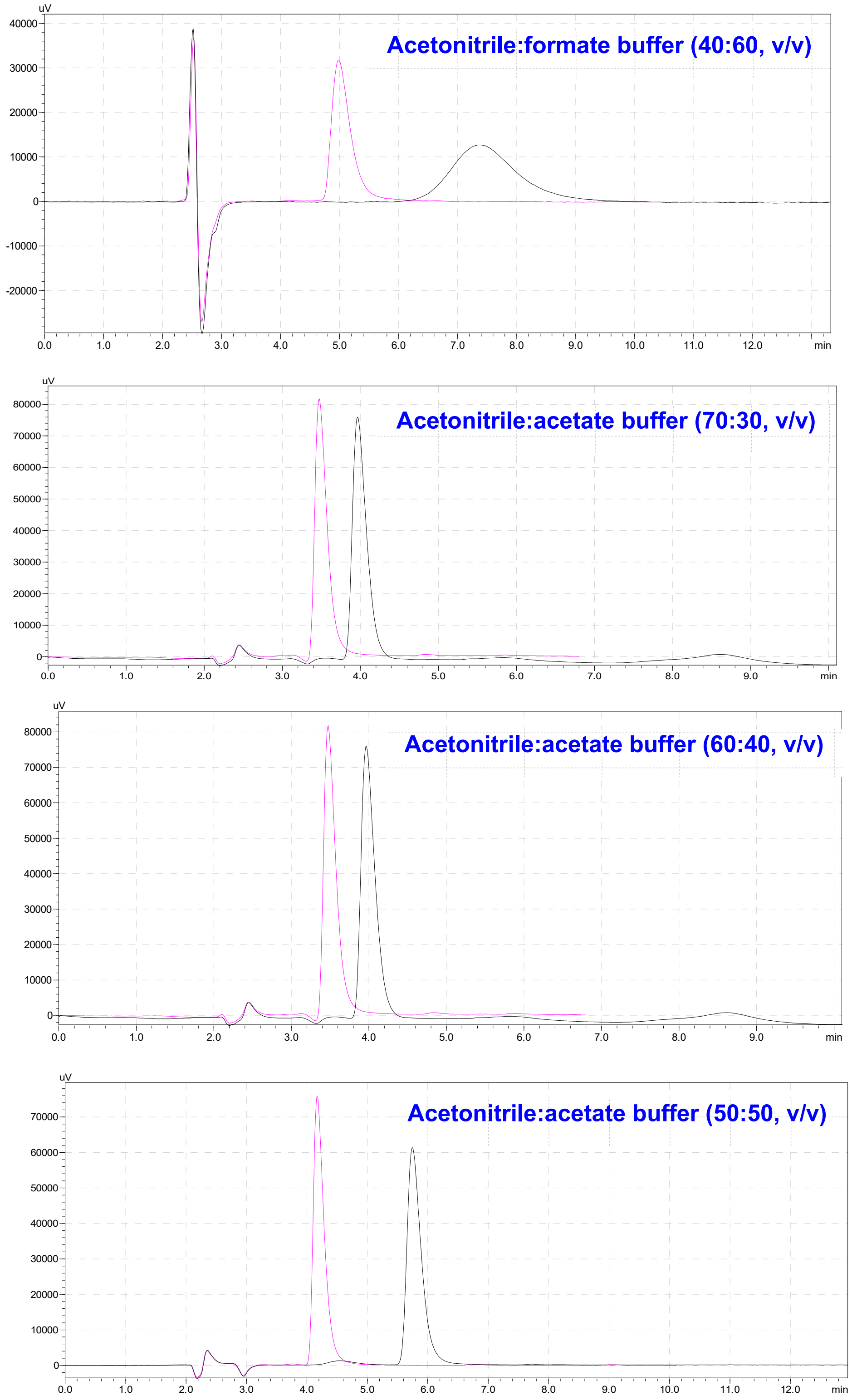

Figure S2: Chromatograms obtained for separation of PAC (black) and SEL (pink) a mobile phase composed of acetonitrile:buffer solution (pH 5) at different ratios. The injected concentrations of PAC and SEL were 10 and 50 µg mL^{-1}, respectively.

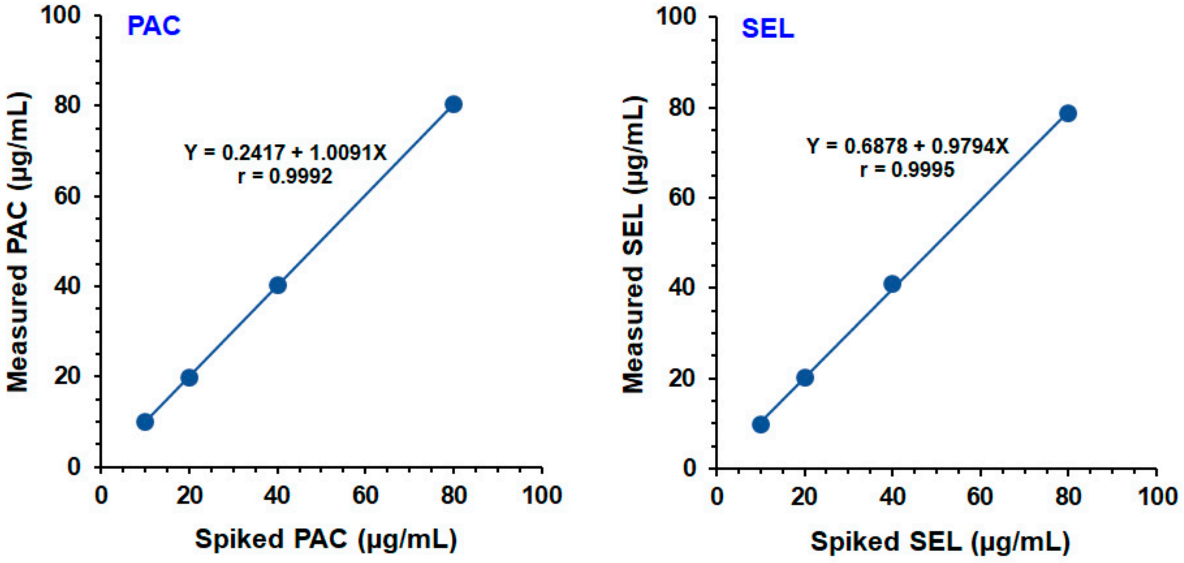

Figure S3: The correlations of concentrations of PAC and SEL spiked in plasma samples with those measured by the proposed HPLC-PDA method. The linear fitting equations and their correlation coefficients are given on the correlation lines.
